# Proceedings of the 1st Symposium “Autoimmune Diseases: Clinical Unmet Needs in Systemic Autoimmune Diseases Guide Clinical, Translational and Basic Research”

**DOI:** 10.31138/mjr.121124.pts

**Published:** 2024-12-31

**Authors:** Loukas Chatzis, Alexandra Koutsogianni, Panagiota Palla, Dimitrios Palamidas, Panagiotis Panagopoulos, Papadaki Maria, Evangelos Andreakos, Athanasios G. Tzioufas

**Affiliations:** 1 Pathophysiology Department, Athens School of Medicine, National and Kapodistrian University of Athens, Athens, Greece,; 2 Research Institute for Systemic Autoimmune Diseases, Athens, Greece,; 3 Laboratory of Immunobiology, Centre for Clinical, Experimental Surgery and Translational Research, Biomedical Research Foundation of the Academy of Athens, Athens, Greece

**Keywords:** meeting report, autoimmunity, clinical research, translational research, basic research

## INTRODUCTION

On October 12–13, 2024, a symposium entitled ‘Autoimmune diseases - Clinical unmet needs in Systemic Autoimmune Diseases guide Clinical, Translational and Basic Research’, was organised by the Research Institute for Systemic Autoimmune Diseases with the scientific contribution of the Medical School of the University of Athens and the Biochemical Research Foundation of the Academy of Athens. This event, chaired by Professor Athanasios G. Tzioufas and Dr. Evangelos Andreakos, was held under the auspices of the Medical School of the National and Kapodistrian University of Athens. The symposium drew 150 participants, including physicians, biologists, and researchers at various academic levels, from professors to students. Renowned Greek and international experts presented recent findings related to systemic autoimmune diseases. The presentations are summarised below, grouped by scientific field.

## SYSTEMIC LUPUS ERYTHEMATOSUS

Rheumatology has witnessed a number of pivotal treatment breakthroughs that have significantly transformed patient care and prognosis. One notable example was Felix Hoffman's development of acetylsalicylic acid (aspirin) at Bayer in 1899 to relieve his father's rheumatoid arthritis pain. A similarly groundbreaking moment occurred in the mid-20th century when Philip Hench at the Mayo Clinic administered “Compound E” to a patient severely affected by rheumatoid arthritis.^1^ Remarkably, within days, the patient experienced profound relief and was able to resume normal activities. Compound E was later named cortisone. More recently, the development of tumour necrosis factor (TNF) inhibitors marked another leap forward. These biologics revolutionised the treatment landscape for a wide range of rheumatic diseases, establishing a new era of biologic therapies.^2^ Today, we may be on the cusp of another transformative shift with the advent of Chimeric Antigen Receptor (CAR) T-cells in rheumatology. The publication of two case series in The New England Journal of Medicine signals towards this direction.^3,4^ Professor Georg Schett has spearheaded this innovative therapy, utilising CAR T-cell technology to harness the immune system's ability to target and eliminate B-cell lineages. His impeccable lecture provided valuable insights into the intricacies and potential of this promising treatment approach for severe autoimmune diseases.

Professor Georg Schett’s lecture began with an engaging overview of the transformative journey in rheumatologic treatments, emphasising the shift from basic symptom relief to targeted, organ-based therapeutic approaches. This advancement was followed by the development of signature cytokine-based concepts, culminating in a forward-thinking vision aimed at achieving disease eradication. Schett proceeded to detail the foundational principles behind CAR T cell creation and function, describing them as “living serial killer cells” that offer superior and deeper B cell tissue depletion compared to conventional anti-CD20 antibodies.^5^ He highlighted the high-level responses observed in patients undergoing CAR T cell therapy, showcasing significant clinical and serologic improvements maintained for at least two years post-treatment. Notable responses were demonstrated across conditions such as systemic lupus erythematosus (SLE), myositis, and scleroderma. Schett then transitioned from clinical outcomes to the underlying mechanisms that contribute to the sustained remission observed in patients. A key factor appears to be the profound and rapid depletion of B cells, with near-complete abrogation occurring within 3 to 7 days post-treatment. This is followed by a reset of naïve B cell populations approximately 100 days later, likened to a “deep, narrow valley” in the immune landscape, signalling the potential for long-term disease control. He further explained that vaccination responses are preserved due to the absence of CD19 on plasma cells. Schett also discussed potential applications of various CARs, such as CD19 and BCMA, within rheumatology. He also addressed the safety and tolerability of CAR T cell treatments, noting that, thus far, no major limitations have emerged. Concluding the session, he presented an inspiring case of a patient with refractory neuro-lupus who experienced significant clinical improvement, suggesting that CAR T cells may effectively penetrate and act within the central nervous system as well.

This captivating lecture sparked a series of compelling questions from the audience that deepened our understanding of CAR T cell implications within rheumatology. Professor Schett offered thought-provoking insights, especially regarding the potential applications of CAR T cell therapy in Sjögren’s Disease and other autoimmune disorders. Given the disease’s plasma cell-driven nature, Schett suggested that BCMA (B-cell maturation antigen) could be a promising target for CAR T cell development. He also discussed potential applications for this therapy in cases of rituximab-refractory granulomatosis with polyangiitis and IgG4-related disease, expanding the scope of its possible impact, while mild diseases and disease accruing a lot of damage might not be the best disease candidates. Additionally, Schett addressed concerns stemming from recent reports in the haematology field about the emergence of T cell lymphomas following CAR T cell therapy providing a reassuring perspective while he suggested that regular vaccinations could be implemented before the conditioning phase of the CAR T cell therapy.^6^

Professor’s Aringer lecture focused on the classification criteria of SLE stressing, that while effective, they are meant for classification, not diagnosis. The goals for such criteria include forming homogenous groups, using feasible and objective measures, and achieving high sensitivity and specificity. The 2019 EULAR/ACR criteria in which Prof Aringer was the lead author aimed to improve early SLE detection and outperform previous versions in accuracy.^7^ These criteria require a positive (ANA) at a titre of ≥ 1:80 as an entry criterion, followed by additive criteria across seven clinical and tree immunological domains, each scored from 2 to 10 points. A score of 10 classifies a patient as having SLE. He emphasised that we should be aware of ANA quality issues and stressed not counting a criterion if another cause is more plausible. External validation showed an ANA sensitivity of 97%, overall criteria sensitivity of 93%, and specificity of 92%, with strong performance in paediatric and early SLE.^8^ Prof Arigner concluded by noting that many Phase 3 RCTs now use the EULAR/ACR criteria.

Professor Bertsias' lecture focused on remission and low disease activity in SLE, highlighting that SLE often involves fluctuating periods of activity and quiescence, with better outcomes linked to achieving low disease activity or remission. First, he discussed multiple targetable factors impacting SLE prognosis, including persistent disease activity, corticosteroid use, presence of anti-phospholipid antibodies, major organ involvement, and comorbidities. He emphasised on two scores, Lupus low disease activity state (LLDAS) and DORIS remission that have been extensively validated against a variety of outcomes and represent quantifiable targets in SLE management. The second part of Professor’s Bertsias lecture was about the concept of “treat-to-target” in SLE. Recent publications have shown that at least 3 to 6 months of low disease activity is beneficial but sustained achievement of the targets is ideal.^9^ A recent study identified three clusters of disease activity over time, with patients showing lower sustained DORIS/LLDAS attainment in cases involving arthritis, mucocutaneous, and neurological symptoms.^9^ Achieving these targets correlates with the reversal of inflammatory changes in PBMCs. Novel biological agents are important add-ons that facilitate the achievement of treatment targets and their sustainability. Novel therapies like belimumab and anifrolumab have shown promise in improving remission and LLDAS rates.^10^ However, he highlighted challenges such as unclear target definitions, organ-specific versus generic targets, monitoring requirements, and limited data for treatment adjustments when targets aren't met. He concluded that, at present, the goal should be clinical remission or low disease activity, facilitated by targeted therapies, while strategies to increase DORIS/LLDAS attainment beyond 60% are still needed.

Professor Fanouriakis’ lecture was about SLE management principles: early diagnosis, treatment aimed at remission or low disease activity, hydroxychloroquine for all patients, minimising steroid use, and employing immunosuppressants or biologics to manage the disease and enable steroid tapering. He highlighted that organ damage risk rises with prednisone doses ≥7.5 mg/day, and reducing glucocorticoids beyond that limit lowers this risk.^11^ Furthermore, according to the latest EULAR recommendations, in patients unresponsive to HCQ (alone or in combination with GC) or those unable to reduce GC doses to safe levels, addition of immunomodulating/immunosuppressive agents (for example methotrexate, azathioprine, or mycophenolate) and/or biologic agents (for example belimumab or anifrolumab) should be considered. A key discussion point was the timing of biologic use—whether early or later in treatment—and their potential to alter SLE's natural course more effectively than conventional drugs.^12^ Recent data demonstrate earlier use of biologic agents over the years and better rates of achievement of targets of therapy such as reduction of disease activity, steroid use, flares and damage and improvement in remission/LLDA and quality of life. The most important point is to select those patients that are candidates for early treatment with biologics and prioritise those who are at high risk to develop severe manifestations. Recent publications have reported childhood onset sle, young adults, males, active serology, moderate to severe lupus with need to use steroids at starting doses of at least 15mg/day and flare upon steroid tapering or after discontinuation of steroids as major risk factors to develop severe lupus manifestations.^13^ Anifrolumab’s efficacy in skin disease was also noted.^14^ The next part of his lecture was about lupus nephritis treatment. Patients with active proliferative lupus nephritis should receive low-dose (EuroLupus) intravenous cyclophosphamide or mycophenolate and GCs, along with belimumab or calcineurin inhibitors (especially voclosporin or tacrolimus, combined with mycophenolate).^13^ Nephroprotection is crucial alongside immunosuppression for long-term care. Combination therapies, while needing more data on patient selection, are approved for active lupus nephritis. The lecture concluded with future therapies, including anti-CD40L, anti-CD20, JAK-STAT inhibitors, and CAR T-cell therapy for refractory SLE.^15,16^

## VASCULITIS

Professor Carlo Salvarani categorised the large vessel vasculitides based on their clinical phenotype and analysed the current treatments available. Glucocorticoids are still the treatment of choice in Giant Cell Arteritis (GCA) as they quickly suppress the inflammation and therefore prevent ischemic complications of the disease.^17^ The necessary duration of GC therapy is variable, but in most patients, it can be discontinued within 1–2 years. However, their prolonged use is associated with several adverse events, creating the need for alternative steroid-sparing therapies to control the disease. Tocilizumab (an IL-6 inhibitor) was introduced in the therapy of GCA after the discovery that IL-6 is strongly implicated in GCA pathogenesis. Current guidelines recommend that all patients with newly diagnosed or relapsing GCA should be treated with tocilizumab. Although the optimal duration of treatment with tocilizumab is unclear, data from 2 clinical studies,^18,19^ suggest that a dose reduction or an increase in the dosing interval in patients in remission after 12 months of TCZ maintains most patients in remission, is safe and cost effective. However, the most worrisome complication of large vessel vasculitis, that is the development of vascular aneurysm cannot be restrained despite the efficient standard of care with TCZ and GCs. Studies have shown that there is a silent vascular progression over time, although patients may be in complete clinical and laboratory remission and there is complete absence of FDG vascular uptake at 2 follow-up PET/CT. The residual vascular inflammation is believed to cause chronic vascular damage as suggested by the data of sequential temporal artery biopsies in Reggio Emilia Hospital (Ricordi et al., RMD Open in press). Finally, 22–33% of the patients may present with aortic dilatations or aneurysms.^20,21^ In the question whether TCZ monotherapy after an ultra-short course of GCs could replace GCs in the treatment of GCA data from GUSTO and TOPAZIO trials were provided leading to the conclusion that the best candidates for TCZ monotherapy might be patients with LVV-GCA without cranial manifestations.^22,23^

Extent discuss was made on the therapeutic options in GCA in cases of TCZ failure. Professor Salvarani provided evidence from randomised control trials (RCTs) that showed positive results of Secukinumab and Upatacitinib. The TiTAIN trial proved that treatment with secukinumab (an IL-17 inhibitor) is a safe and efficient choice in patients with new onset or relapsing GCA naïve to biological therapy.^24^ Regarding the JAK inhibitors in GCA, based on the results of SELECT-GCA trial which showed positive results, their use is recommended by EMA in patients over 65 years only if no suitable treatment alternatives are available.

Professor David Jayne talked about new aspects in the pathophysiology of antineutrophil cytoplasmic antibody (ANCA) associated vasculitis (AAV) and divided his lecture into three parts: immune pathways, molecular targets and implications for future therapy of AAV. First, he discussed the immune pathways participating in AAV pathogenesis. Genetics of AAV associate with ANCA subtype, not clinical diagnosis – proteinase 3 (PR3) specific ANCA associate with HLA-DP alleles and proteinase 3 gene, while myeloperoxidase (MPO) specific ANCA associate with HLA-DQ alleles. Neutrophils play a central role in AAV pathogenesis. Activated neutrophils have increased expression of membrane PR3 and MPO. ANCA bind to membrane PR3 and MPO via their Fab region, while their Fc region binds to FcŞ receptors on the surface of neutrophils, leading to further activation of these cells. Furthermore, ANCA induce neutrophil extra-cellular traps (NET) formation, containing PR3 and MPO autoantigens and contributing to neutrophil mediated tissue injury. Furthermore, the alternative complement pathway participates in AAV pathogenesis. ANCA bind to neutrophils and activate alternative complement pathway, leading to C3a, C5a production, which perpetuate neutrophil activation, creating a vicious cycle. Recent publications have reported C3, C4 deposition in glomeruli and arterioles in AAV and association of MPO-ANCA affinity with complement activation.

The second part of Professor Jayne’s lecture was about molecular targets for the treatment of AAV. A recent study reported elevated serum levels of B-cell activation factor of the TNF family (BAFF) and a proliferation-inducing ligand (APRIL) in AAV patients. Lower expression of BAFF receptor on memory B cells and higher expression of TACI receptor on CD19+ cells and immature B cells were also demonstrated in patients with AAV. Alternative complement pathway is a known molecular target in AAV and may be inhibited by the C5a receptor antagonist avacopan(25). Recently, it has been reported that plasma and urine levels of Factor Ba rise with kidney flare in AAV. Furthermore, common genetic variants in Factor B, Factor H and Factor H receptor are associated with disease susceptibility or severity of kidney damage in AAV.

In the final part of his lecture, Professor Jayne discussed future therapies for AAV, outlining limitations of current treatments like rituximab, which include high relapse rates, infection risks, secondary immunodeficiency, and insufficient depletion of B cells at the level of the tissue.^26^ Newer B cell therapies are being proposed, targeting: B cell depletion (Obinutuzumab, Anti-CD19 CAR-T), B cell activation (Alemtuzumab, CTLA4/Abatacept) and B cell cytokines (BLyS/Belimumab, BLyS/APRIL: Atacicept, Povitacicept, Telitacicept, Anti-APRIL: Sibeprenlimab, Anti-BAFF receptor: Ianalumab). Obinutuzumab (anti-CD20) is more effective than rituximab at depleting tissue B cells and limited data from trials suggest that use of anti-BAFF/APRIL agent could prove useful in the management of AAV. Regarding complement targeting, ADVOCATE trial has demonstrated that avacopan, a C5a receptor inhibitor, was noninferior but not superior to prednisone taper with respect to remission at week 26 and was superior to prednisone taper with respect to sustained remission at week 52.^27^ Avacopan also led to faster proteinuria reduction and better kidney function recovery. Ongoing trials are exploring anti-C5a and anti-factor B treatments for AAV. Finally, lixudebart lixudebart, an anti-Claudin-1 monoclonal antibody aimed at reversing fibrosis, is being tested in a Phase 2 trial for Rapidly Progressive Glomerulonephritis AAV patients. Prof. Tzioufas’ presentation examined the cellular drivers of tissue injury in GCA, a primary form of Large Vessel Vasculitis (LVV). The presenter elegantly highlighted the significance of PET/CT imaging and biopsy data in revealing key disease mechanisms and biomarkers.^28^ The role of macrophages, dendritic cells, and key cytokines like IL-6 and GM-CSF are critical to disease progression, and complications such as aneurysm formation were also underlined.^29^

Focus was given on cellular senescence and the senescence-associated secretory phenotype (SASP) in the pathogenesis of GCA. Histopathological analyses in GCA patients revealed distinct features of cellular senescence, including the accumulation of lipofuscin granules and elevated levels of p16 and p21 markers. Inflammatory cell types such as senescent fibroblasts, macrophages, and vascular smooth muscle cells (VSMCs) were identified in GCA lesions, predominantly in areas of transmural inflammation.^30^ Comparison with control samples from polymyalgia rheumatica (PMR) patients showed significantly higher SASP expression, with an increase in IL-6-positive senescent cells in GCA tissues. Furthermore, MMP-9 was notably elevated in GCA, suggesting its role in extracellular matrix degradation and arterial remodelling.^30^

Experimentally, GCA supernatant was shown to induce senescence in primary skin fibroblasts, VSMCs, and human umbilical vein endothelial cells (HUVECs), increasing SASP markers like IL-6 and MMPs, which are instrumental in inflammation and tissue restructuring. Tocilizumab, an IL-6 receptor blocker, partially inhibited SASP induction in these cells, providing evidence for IL-6’s central role in maintaining senescence and inflammation in GCA. Co-staining techniques further demonstrated the presence of GL13/IL-6/p21WAF1/Cip1 in senescent cells, highlighting IL-6 as a driver of persistent vascular cell senescence.^30^

The presentation also parallels findings in Takayasu's arteritis, another large vessel vasculitis, where vascular smooth muscle cell (VSMC) senescence—driven by IL-6–STAT3 signalling—is implicated in the underlying inflammatory response.^31^ These findings underscore the role of IL-6-positive senescent cells in vascular injury across LVV types and highlight their potential as a therapeutic target.

Prof. Tzioufas’ presentation outlined unresolved questions about the mechanistic role of senescence in GCA, particularly regarding the formation of vascular aneurysms and residual tissue inflammation after acute inflammatory phases. The presentation concludes with recommendations for future research to refine treatment selection tools, with a focus on biomarkers specific to long standing inflammation generating structures such as senescent cells with a SASP signature.

Dr. Constantinos Anagnostopoulos presented on the application of 18F-FDG PET/CT imaging for evaluating inflammation in the vascular territories, with a specific focus on LVV. PET/CT imaging as a highly sensitive method, enables the early detection of inflammatory activity across large vessels and the quantitative measurement through the target-to-background ratio (TBR) method.^32^ This imaging approach is particularly valuable for diagnosing LVV and assessing vascular inflammation in various chronic conditions, such as atherosclerosis, rheumatoid arthritis, and systemic lupus erythematosus. However, this imaging technique has certain limitations, including high costs and radiation exposure risks, highlighting the importance of collaboration between nuclear medicine specialists and clinicians for optimal patient outcomes.^33^

In various studies, FDG uptake on PET/CT was correlated with a higher risk of cardiovascular events, including aortic aneurysms and major adverse cardiovascular events (MACE).^34^ This association is particularly evident in diseases with systemic inflammation, such as GCA, where PET/CT imaging can indicate increased vascular inflammation.^35^ Additionally, in conditions like psoriasis, PET/CT has linked inflammation severity with elevated cardiovascular risks, reinforcing the value of PET/CT in risk stratification for patients with chronic inflammatory disorders.^36^

Monitoring treatment efficacy in these cases presents its challenges. Persistent FDG uptake in vascular tissues after treatment can reflect either active inflammation or the remodeling process of blood vessels. This ambiguity makes it essential to carefully interpret PET/CT results to avoid misclassifying disease activity. The method has proven beneficial in detecting subclinical inflammation in patients without overt cardiovascular symptoms, including those with chronic inflammatory diseases like HIV and systemic lupus erythematosus.^37^

Emerging innovations in PET tracers are expanding the utility of PET/CT. New tracers, such as those targeting somatostatin receptors and CXCR4, show promise in enhancing specificity by differentiating inflammation types, which could help distinguish between vasculitic and atherosclerotic inflammation.^38^ Additionally, artificial intelligence is being explored to enhance the reliability of PET/CT analyses by standardising the assessment of radiotracer uptake across different vascular regions, potentially leading to more accurate diagnoses and treatment assessments.^39^

In summary, 18F-FDG PET/CT imaging is a valuable tool in managing vascular inflammation, particularly in complex, chronic inflammatory diseases. As imaging technology continues to advance, multidisciplinary collaboration among healthcare providers will be crucial in leveraging these innovations to improve diagnostic precision and treatment monitoring. The integration of new tracers and AI-driven analytics holds potential for future improvements in accurately assessing disease activity and providing targeted, individualised patient care.

## INTERSTITIAL LUNG DISEASE IN RHEUMATOLOGY

Professor Oliver Distler talked about interstitial lung disease (ILD) in rheumatic and musculoskeletal diseases (RMD), including systemic sclerosis (SSc), rheumatoid arthritis (RA), idiopathic inflammatory myopathies (IIM), mixed connective tissue disease (MCTD), Sjögren’s disease (SjD) and SLE. First, he talked about prevalence and screening of ILD in RMDs. Patients with SSc, IIM and MCTD demonstrate a high prevalence of ILD (40–60%), RA, SjD patients have a lower prevalence of ILD (10–20%) and SLE is rarely associated with ILD.^40^ All patients with SSc, MCTD and IIM should be screened for ILD at baseline, with high resolution tomography (HRCT) of the lungs. Interestingly, applying the risk factors for the development of ILD in SSc, described the ACR 2023 recommendations for SARD-ILD screening, would miss 25% of SSc patients with ILD.^41,42^ Concerning screening of ILD in RA, Narvaez et al. have proposed screening criteria based on Delphi methodology.^43^ ILD in RMD may present after disease diagnosis and the annual incident rate of new ILD in SSc is 3.8 per 100 person-years. Therefore, some patients may benefit from screening for ILD in follow-up visits. Then, Professor Distler went on to discuss monitoring of ILD in RMDs, which should be done using pulmonary function tests (PFTs) regularly and HRCT as needed. SSc-ILD and RA-ILD may progress (meaning decline of FVC and/or radiological disease progression and/or worsening of respiratory symptoms) even within a month and even minor ILD progression in SSc is associated with mortality. ILD progresses in 5–30% of RMD patients, the proportions varying for each RMD. Progression starts early and continues even into late stages of SSc-ILD, while significant progression of SSc-ILD happens in discrete phases. Importantly, a significant proportion of SSc-ILD patients who die of respiratory causes have mild lung disease. 75% of respiratory deaths in patients with preserved lung function are caused by respiratory tract infections. Professor Distler went on to discuss the treatment of RMD-ILD in the final section of his lecture. Theurapeutic options for RMD-ILD include rituximab, mycophenolate, cyclophosphamide and nintedanib, while tocilizumab is another option for SSc-ILD.^44^ Level of evidence is important when selecting a drug for RMD-ILD. The phenotype of the RMD-ILD patient is another important factor for selecting therapeutic agent and ideally it should fit the inclusion criteria of the clinical trial of the respective agent, if there is one.

## SJÖGREN'S DISEASE

Professor Goules analysed in his lecture the predictors of lymphomagenesis in Sjögren’s disease. Sjögren’s disease exhibits a higher prevalence of lymphoma compared to other autoimmune diseases. The most common lymphoma associated with Sjögren’s is MALT (Mucosa associated lymphoid tissue) lymphoma followed by nodal marginal zone lymphoma (NMZL) and DLBCL.^45^

The lymphomagenesis predictors in SjD involve several clinical and biological characteristics such as salivary gland enlargement, palpable purpura, cryoglobulinaemia, hypocomplementaemia, etc.^46^ Patients with SjD can be stratified for the risk of lymphoma development according to these features in two categories. As high or low risk. Professor Goules also exhibited the clinical features from 7551 retrospectively harmonised patients with SjD selected from a European patient cohort.^47^ This database provides interesting clinical information about the lymphoma associated risk factors. Lymphomagenesis is a multistep process involving the B cell component that is mainly taking place in the context of the ectopic Germinal Centre-like structures.^48^ Initially, RF and IgM B cell are expanding through an antigen selection process under the effect of growth factors, cytokines that gradually leads to oligoclonal/monoclonal expansion and malignant transformation after mutations of oncogenes or tumor suppressor genes of the germline DNA. Previous studies on lymphoma predictors included relatively small cohorts, had typical logistic regression models and did not take into consideration the duration from SjD to lymphoma diagnosis. The aforementioned cons created the need of a new study that enrolled patients from 3 centers specialised in SjD and aimed to identify MALT lymphoma predictors in Sjögren’s disease, present at the timepoint of SjD diagnosis and 3–4 years before the diagnosis of MALT lymphoma.^49^ According to the results of the study i) cryoglobulinemia and salivary gland enlargement imply either an already existing lymphoma or high risk for future lymphoma development, ii) Rheumatoid Factors are the most persistent and chronically distant MALTL predictor, iii) The addition of cutaneous, glandular, hematologic and biologic manifestations define an advanced time point of MALT lymphomagenesis, iv) Systemic clinical manifestations and especially cryoglobulinemic can be attributed to both the disease itself or an underlying lymphoma, and finally SjD-MALTLs evolve slowly and patients should undergo lip biopsy at the time of diagnosis.^49^

Dr Chatzis in his lecture talked about current and future possible treatments of Sjögren’s disease based on new clinical studies. Treatment of SjD remains a challenge, as most of the therapies target systemic manifestations more effectively, while the glandular symptoms are clearly more difficult to manage.^50^ A reason for that might be the complex pathophysiology of the disease involving both B and T lymphocytes that collaborate to form germinal centers promoting immunoglobulin class switching and the production of high affinity autoantibodies. Many randomised trials failed to demonstrate treatment efficacy partly because their outcomes were not validated.^51^ However, newer studies focusing on specific disease subpopulations and having set a specific validated primary endpoint such as the change in ESSDAI, provided the first positive results. Dr. Chatzis also discussed the evolution in trial design, noting the use of umbrella studies with two mutually exclusive treatment arms to enhance inclusivity. Dr Chatzis triggered by the pathophysiology of the disease analysed the results of large clinical trials that showed promising results. Ianalumab, iscalimab, remibrutinib, dazodalibep are some of the targeted treatments that met their primary endpoints and could therefore serve as a possible treatment option in the future.^52–55^ In conclusion the targeting of specific SjD pathways and improved outcome measures able to capture the Sjögren’s complexity may provide us with more appropriate and innovative treatments in the future. For the time being, treatment of some extraglandular symptoms and SjD associated symptoms of dryness and fatigue remains an unmet medical need.

## AUTOINFLAMMATORY DISEASES

Professor Sfikakis' lecture on TNF inhibitors in Behçet’s Disease (BD) was divided into four parts: introduction, history, current experience, and future proposals. First, he covered BD’s epidemiology, noting its prevalence along the historical Silk Road, and its clinical features, including skin lesions, recurrent oral and genital ulcers, ocular, CNS, intestinal, joint inflammation, and vascular involvement. The 1990 ISBD classification criteria were discussed, with a note that HLA-B51 is not recommended for diagnosis. In the second part of his speech, he referred to the history on TNF inhibitors in BD. He reviewed early use cases, highlighting a 2001 study where infliximab successfully treated sight-threatening panuveitis in patients on a regimen of prednisone, azathioprine, and cyclosporine. Subsequent studies confirmed the safety and efficacy of TNF inhibitors for refractory ocular inflammation unresponsive to standard treatments. In the third part of his lecture, he detailed TNF inhibitors' broad efficacy across various BD manifestations. A recent study reported high response rate of 81% with the use of infliximab in refractory uveoretinis with simultaneous reduction in CsA and CS co-administration and a good safety profile. A meta-analysis revealed an 85% ocular inflammation remission rate among uveitis patients treated with TNF inhibitors and a 67.3% retention rate for anti-TNF during follow-up. Adalimumab showed slight superiority among TNF inhibitors. Furthermore, TNF inhibitors were found safe and effective in BD patients with intestinal manifestations, vascular involvement and chronic progressive Neuro-Behçet’s disease. Comparatively, infliximab induction therapy proved better and safer than cyclophosphamide for severe BD cases. Early initiation of TNF inhibitors for uveitis was linked to better outcomes. According to recent publications, de novo manifestations were infrequent among BD patients treated with TNF inhibitors. Long-term use allowed for potential drug-free remission over five years, and withdrawal after two successful treatment years was suggested, though relapses occurred in about 10% of cases that do not respond to anti-TNF re-treatment. In the last part of his lecture, Professor Sfikakis proposed that patients with non-mucocutaneous/arthritic manifestations should be treated with anti-TNF monoclonal antibodies as first-line therapy, as early as possible. Patients achieving two years of remission could consider discontinuation with close monitoring. Non-response to TNFi indicated a difficult-to-treat status, necessitating alternative biologics He argued that placebo-controlled RCTs for severe BD manifestations are unethical and that new drugs should be benchmarked against adalimumab or infliximab without a placebo arm. Finally, Prof Sfikakis concluded that recommendations for a treat-to-target strategy in BD are needed, and further studies should assess whether anti-TNF treatments have reduced BD mortality.

Professor Skendros presented the work of his laboratory and the current perspectives on the involvement of neutrophils in autoinflammatory diseases (AIDs). The main clinical symptom of AIDs is fever of unknown origin (FUO), with 21% of FUO patients obtaining a final diagnosis of an autoinflammatory or autoimmune disease.^56^ The common characteristics of AIDs are periodic or chronic systemic inflammation, dysregulation of innate immunity, absence of autoantibodies and autoreactive T cells, and absence of active infection. AIDs can be monogenic diseases [Familial Mediterranean Fever (FMF), Cryopyrin-associated autoinflammatory syndromes (CAPS), TNF receptor-associated periodic fever syndrome (TRAPS), etc.] or polygenic/multifactorial diseases (Adult-onset Still's disease, Behcet disease, etc.).^57^ Prof Skendros presented the latest classification criteria for autoinflammatory recurrent fevers^58^ and referred to newly characterised autoinflammatory syndromes uncovered by cutting-edge Next Generation Sequencing methodologies, including VEXAS syndrome.^59^ Moving on to the main part of his talk, the involvement of neutrophils and NETs in the pathophysiology of FMF was discussed. During acute inflammatory attacks in FMF, neutrophils release high amounts of NETs decorated by bioactive IL-1,^60^ and the underlying mechanism of NET release is autophagy-driven.^61^ Taken together, their group suggests a model of IL-1-associated autoinflammation characterised by dysregulated inflammasome activation & autophagy-mediated NETosis as the underlying mechanism of FMF pathogenesis. Finally, Prof Skendros presented his group's latest work, addressing the clinical unmet need for a dependable biomarker to distinguish between active infection and autoinflammatory disease presentation. Natsi et al. suggest an IL-1Ş/DNA complex ELISA approach to distinguish autoinflammatory disorders from autoimmune and infectious diseases suitable for everyday clinical practice.^62^

## CO-MORBIDITIES IN RHEUMATOLOGY

Professor Vassilopoulos in his lecture focused on the infections in ARDs. Patients with autoimmune diseases are particularly prone to infections due to both the underlying immune dysfunction and the use of immunosuppressive therapies.^63^ They may present common bacterial infections as people in general population with common sites being the respiratory and urinary tract as well as the skin and soft tissues. The risk for bacterial infections is determined by the underlying disease, comorbidities and of course the received treatment. Chronic glucocorticoid use is a well-established risk factor for infections and studies have shown that a dose of prednisone equivalent ≤5mg/day is generally safe for use. In rheumatoid arthritis the RABBIT risk score for calculating the risk for infections has been proposed.^64^ Apart from the bacterial infections, the reactivation of underlying viral infections such as chronic hepatitis B is another considerable issue. All patients with ARDs should be screened serologically for hepatitis B before starting immunosuppressive therapy and receive proper antiviral treatment if found with a chronic infection. Depending on the degree of disease activity and immunosuppression patients with ARDs may be particularly prone to opportunistic infections (OIs) as well, which are associated with significant morbidity and mortality.^65^ The most common OIs are from Mycobacterium Tuberculosis and Pneumocystis jirovecii.^66^ Prof Vassilopoulos also highlighted the need for prophylaxis through vaccination against influenza, pneumococcus, herpes zoster, and RSV. Since infections can affect the outcomes of patients with ARDs, careful monitoring and preventing measures play a key role in the everyday clinical setting.

Professor Kitas talked about the cardiovascular risk in Rheumatoid arthritis. In different studies subclinical atherosclerosis appears to be more pronounced in RA compared to controls similarly as in Diabetes mellitus type 2.^67^ Chronic inflammation seems to play a key role in this procedure by affecting the endothelial function. However, classical CVD risk factors such as hypertension, obesity, dyslipidaemia, smoking, etc. have a greater impact than systemic inflammation on most vascular parameters. Regarding the hypertension and dyslipidaemia, they are highly prevalent in patients with RA, often underdiagnosed and amongst others they associate with chronic steroid use, obesity and elevated serum uric acid levels. Overall, high-grade chronic inflammation leads through various pathways in the so-called muscle wasting and cachexia that in association with the physical inactivity in RA changes the body composition and the metabolic profile of the patients.^68^. Classical and novel biologic treatments of RA were discussed separately since they have their own effects on the CVD risk factors. Careful monitoring of individual CVD risk factors in patients with RA will allow early intervention through lifestyle modifications and pharmacological treatment and will eventually lead to a better quality of life.^69^

## CLINICAL IMPLICATIONS OF BASIC RESEARCH

Dr Andreakos discussed about the pleiotropic effects of Type III interferons in inflammation and beyond. As IFNs are strongly related to viral respiratory infections, he pointed out that antiviral immune responses need to be fine-tuned to optimise protection. Otherwise, there is always the risk of excessive cytokine responses (storms) that lead to severe respiratory infections with excessive lung damage. He stated that Type I IFNs are the primary system of antiviral defense, as evidenced by over 60 years of extensive research. In 2003 the role of IFNλs was first described in antiviral protection, through a distinct class II cytokine receptor.^70,71^ He clarified that IFNλs share induction mechanisms and signaling cascades with type I IFNs.^72^ To study the role of IFNλs in the respiratory tract, with his team he developed an IFNλR1 reporter mouse model and showed that IFNλR1 is broadly expressed in the mouse lung and that IFNλs are induced first, specifically by airway epithelial cells, upon flu infection, before type I IFNs. Mechanistically they found that IFNλR1 on both respiratory epithelial cells and neutrophils is required to control initial viral replication and spread. They also found that type I IFNs activate neutrophil pro-inflammatory responses, and prevent systemic spread, whereas type III IFNs act at the barrier surface. He concluded that IFNλs are necessary for preventing overactivation of the innate immune response during infection.^73^ In human studies, Dr Andreakos and his team studied IFNλs in COVID-19 and flu patients and demonstrated that COVID-19 patients exhibit impaired and delayed type I and type III IFN production. IFNλ production in COVID-19 patients is associated with better outcome. A sharp contrast to flu which expresses type I and type III IFNs across the spectrum of disease severity. He concluded that this ‘untuned’ antiviral immunity in COVID-19 may underlie the high incidence of pneumonia and critical illness observed.^74^ Dr Andreakos research significantly contributed to the finding that IFNλ plays a crucial role in viral load and hyperinflammation in COVID-19.^75^ A result further verified with clinical studies with administration of pegylated IFNλ in COVID-19 patients.^76^ In addition to their role in antiviral immunity, there is some evidence suggesting that IFNλs exhibit additional functions beyond antiviral immunity, which remain poorly understood.^77^ For example, in neutrophil-driven thromboinflammation.^78^ Moreover, Dr Adreakos and his team found that IFNλ exhibits a completely unexpected novel function in obesity, metabolic regulation and cardiovascular disease. Specifically, abrogation of IFNλ signaling in mice leads to white adipose tissue inflammation, severe obesity, insulin resistance and metabolic deregulation and severe atherosclerosis (CVD). This occurs under regular chow diets and is exacerbated under high fat diet (HFD). On the contrary, recombinant IFNλ administration in wild type mice reduces adipose tissue and systemic inflammation, treats HFD-induced obesity, metabolic deregylation and atherosclerosis (CVD). Consequently, IFNŞ demonstrates functions that extend beyond antiviral activity and is currently being studied in the context of autoimmune diseases.

Dr Tamvakopoulos addressed the significance of metabolomics in the context of inflammatory conditions. He specifically noted that Mass Spectrometry-based methods enable us to address pharmacokinetics and drug mechanism enquiries, ultimately enhancing targeted drug delivery. His research interests include the identification of bioactive small molecules, peptides, or peptide conjugates, and more recently, PROTACS/Protein Degraders, for the targeted treatment of solid tumors. This strategy takes advantage of aberrant target expression in cancer cells to preferentially target the cancer cells rather than the adjacent healthy tissues. Then, he emphasised on the significance of biomarkers and the fact that they are employed in a variety of scientific disciplines, with numerous uses at various phases of the drug development process. The accuracy of biomarkers can vary, therefore, not all biomarkers are suitable for medicines development. For example, therapeutic interventions may influence biomarkers and clinical endpoints in clinical trials. He provided few well-known examples of biomarkers, such as glucose levels, that are used to manage diabetes. Images of the brain, such as Magnetic Resonance Imaging (MRI), that can be used to determine the progression of Multiple Sclerosis, etc. He also presented new biomarkers that are currently being tested, specifically incretin hormones as biomarkers of diabetes treatment (Weber AE 2004). He then proceeded to discuss a critical aspect of the study of biomarkers and he explained the LC-MS/MS methods that can be established for metabolite quantification and validation studies. Conversely, he also acknowledged the obstacles associated with the utilisation of biomarkers in the development of pharmaceuticals. As the use of biomarkers in pharmaceutical research expands there are several challenges to overcome, including ethical, regulatory, and technical barriers. He went on to discuss the distinction between untargeted and targeted metabolomics studies. Untargeted or discovery-based metabolomics focuses on global detection and relative quantification of small molecules in a sample. In contrast, targeted or validation-based metabolomics focuses on measuring well-defined groups of metabolites with opportunities for absolute quantification.^79^ He provided an example of an untargeted metabolomic study in which the role of deoxyinosine in attenuating collagen-induced arthritis in mice was revealed through LC-MS-based rheumatoid arthritis serum metabolomics.^80^ He then elaborated that his team is in a unique position to participate in targeted studies that are applicable for pharmacokinetic and biomarker efficacy/toxicology studies. This is due to the fact that they are a GLP accredited bioanalytic facility and they use high technology Mass Spectrometry-based technologies. With his team, they used LC-MS/MS approach in TDM to achieve simultaneous quantification of combination therapies in Cancer patients. Very recently, they have also used a LC-MS/MS approach to perform targeted lipidomic analysis and measure Lipid Mediators, compounds that are challenging to measure, as they have low detection limits. Specifically, they were able to develop an analytical LC-MS/MS method for the determination of Lipid Mediators. This method is able to be used with low sample quantity (200 μL) and it has also a quick analysis time (15 mins). With this method they were able to validate a key lipid mediator of vascular inflammation as a biomarker for ICU admission and lung injury in COVID-19 patients (Papadaki et al., 2024, under revision).

Dr Kambas reviewed the roles of neutrophil extracellular traps (NETs) in inflammation and autoimmune diseases. NETs, which are web-like DNA structures released by neutrophils, are induced by triggers such as phagocytosis, IL-8 signaling, and platelet activation through mechanisms involving reactive oxygen species (ROS), myeloperoxidase expression (MPO), and PAD4-mediated histone citrullination.^81^ NETs contribute to inflammatory and autoimmune processes, including thrombosis, cancer, fibrosis, and autoimmunity.^82^ In SLE, NETs provide antigens that stimulate the production of autoantibodies, as well as the release of type I interferons through activation of plasmacytoid dendritic cells.^83^ Impaired clearance of NETs, due to DNase I inhibitory activity, correlates with increased disease severity in SLE.^84,85^ In rheumatoid arthritis (RA), NETs are linked to joint inflammation and tissue damage.^86^ NETs are also implicated in GCA, where they interact with cytokines IL-6 and IL-17A.^87^ Additionally, in antiphospholipid syndrome (APS), anti-β2GPI antibodies induce NET formation, leading to thrombosis.^88^ The provided evidence in this presentation supports the hypothesis that NETs are a unifying pathogenic mechanism in various inflammatory and thrombotic conditions, underscoring their potential as therapeutic targets.

Dr Galani discussed the application of spectral flow cytometry in characterising immune responses in autoimmune and autoinflammatory diseases. Compared to conventional flow cytometry, Spectral cytometry offers advanced detection capabilities by using continuous narrow bandpass filters that allow the discrimination of spectra with significant overlap, thus enhancing the scope of immunophenotyping.^89^ This method enables deeper analysis of immune cells across tissues and blood samples.

The presenter showcased experimental protocols for mouse and human samples, including a 19-color panel for profiling adipose-tissue macrophages in obesity and 15-color panels for characterising B cell subsets in murine bone marrow and spleen.^90^ Additionally, her group is implementing a spectral cytometry protocol for assessing immune cell populations in the blood of patients with autoimmune conditions such as GCA, ANCA Vasculitis, and PMR. Unpublished findings reveal distinct alterations in immune cell compositions across these conditions, including reduced T and NK cells in GCA and ANCA vasculitis, increased CD8+ T cells in ANCA vasculitis, and differential levels of neutrophils and monocytes. The establishment of these multi-color panels underscores the potential of spectral cytometry to advance understanding of immune dysregulation in autoimmune diseases.

## DISCUSSION

The first collaborative meeting between the Research Institute for Systemic Autoimmune Diseases and the Biochemical Research Foundation of the Academy of Athens successfully created an amalgam of clinical expertise with deep insights into the pathogenesis of immune system alterations. This symposium featured clinical, translational, and basic research perspectives presented by leading experts in the field, fostering a comprehensive understanding of systemic autoimmune diseases. The event's significant attendance highlighted the enthusiasm within the scientific community and generated considerable anticipation for the second meeting, underscoring its potential as a key annual gathering for advancing autoimmune disease research and collaboration.

## References

[1] Hench PS Kendall EC Slocumb CH Polley HF. The effect of a hormone of the adrenal cortex (17-hydroxy-11-dehydrocorticosterone: compound E) and of pituitary adrenocortical hormone in arthritis: preliminary report. Ann Rheum Dis 1949;8(2):97–104. 18623812 10.1136/ard.8.2.97 PMC1030685

[2] Feldmann M Maini RN. Lasker Clinical Medical Research Award. TNF defined as a therapeutic target for rheumatoid arthritis and other autoimmune diseases. Nat Med 2003;9(10):1245–50. 14520364 10.1038/nm939

[3] Mougiakakos D Krönke G Völkl S Kretschmann S Aigner M Kharboutli S CD19-Targeted CAR T Cells in Refractory Systemic Lupus Erythematosus. NEJM 2021;385(6):567–9. 34347960 10.1056/NEJMc2107725

[4] Müller F Taubmann J Bucci L Wilhelm A Bergmann C Völkl S CD19 CAR T-Cell Therapy in Autoimmune Disease - A Case Series with Follow-up. NEJM 2024;390(8):687–700. 38381673 10.1056/NEJMoa2308917

[5] Schett G Müller F Taubmann J Mackensen A Wang W Furie RA Advancements and challenges in CAR T cell therapy in autoimmune diseases. Nat Rev Rheumatol 2024;20(9):531–44. 39107407 10.1038/s41584-024-01139-z

[6] Hamilton MP Sugio T Noordenbos T Shi S Bulterys PL Liu CL Risk of Second Tumors and T-Cell Lymphoma after CAR T-Cell Therapy. NEJM 2024;390(22):2047–60. 38865660 10.1056/NEJMoa2401361 PMC11338600

[7] Aringer M Costenbader K Daikh D Brinks R Mosca M Ramsey-Goldman R 2019 European League Against Rheumatism/American College of Rheumatology classification criteria for systemic lupus erythematosus. Ann Rheum Dis 2019;78(9):1151–9. 31383717 10.1136/annrheumdis-2018-214819

[8] Aringer M Petri M. New classification criteria for systemic lupus erythematosus. Curr Opin Rheumatol 2020;32(6):590–6. 32925250 10.1097/BOR.0000000000000740 PMC8020886

[9] Pitsigavdaki S Nikoloudaki M Garantziotis P Silvagni E Repa A Marangoni A Pragmatic targets for moderate/severe SLE and their implications for clinical care and trial design: sustained DORIS or LLDAS for at least 6 months is sufficient while their attainment for at least 24 months ensures high specificity for damage-free progression. Ann Rheum Dis 2024;83(4):464–74. 38233103 10.1136/ard-2023-224919 PMC10958283

[10] Bertsias G Yazdany J. Achievable but elusive: LLDAS and DORIS remission in clinical trials of belimumab. Lancet Rheumatol 2024;6(11):e734–e5. 39208826 10.1016/S2665-9913(24)00231-5

[11] Al Sawah S Zhang X Zhu B Magder LS Foster SA Iikuni N Effect of corticosteroid use by dose on the risk of developing organ damage over time in systemic lupus erythematosus-the Hopkins Lupus Cohort. Lupus Sci Med 2015;2(1):e000066. 25861455 10.1136/lupus-2014-000066 PMC4378372

[12] Fanouriakis A Kostopoulou M Andersen J Aringer M Arnaud L Bae SC EULAR recommendations for the management of systemic lupus erythematosus: 2023 update. Annals of the rheumatic diseases. 2024;83(1):15–29. 37827694 10.1136/ard-2023-224762

[13] Katechis S Nikolopoulos D Flouda S Adamichou C Chavatza K Kapsala N Can we predict kidney involvement in patients with systemic lupus erythematosus? A retrospective cohort study with independent validation. Rheumatology (Oxford, England) 2024. 10.1093/rheumatology/keae278 38745434

[14] Flouda S Emmanouilidou E Karamanakos A Koumaki D Katsifis-Nezis D Repa A Anifrolumab for systemic lupus erythematosus with multi-refractory skin disease: A case series of 18 patients. Lupus 2024;33(11):1248–53. 39098049 10.1177/09612033241273023

[15] Merrill JT Tanaka Y D'Cruz D Vila-Rivera K Siri D Zeng X Efficacy and Safety of Upadacitinib or Elsubrutinib Alone or in Combination for Patients With Systemic Lupus Erythematosus: A Phase 2 Randomized Controlled Trial. Arthritis Rheumatol (Hoboken, NJ) 2024;76(10):1518–29. 10.1002/art.42926 38923871

[16] Mackensen A Müller F Mougiakakos D Böltz S Wilhelm A Aigner M Anti-CD19 CAR T cell therapy for refractory systemic lupus erythematosus. Nat Med 2022;28(10):2124–32. 36109639 10.1038/s41591-022-02017-5

[17] Dejaco C Kerschbaumer A Aletaha D Bond M Hysa E Camellino D Treat-to-target recommendations in giant cell arteritis and polymyalgia rheumatica. Ann Rheum Dis 2024;83(1):48–57. 36828585 10.1136/ard-2022-223429 PMC10803996

[18] Calderón-Goercke M Loricera J Moriano C Castañeda S Narváez J Aldasoro V Optimisation of tocilizumab therapy in giant cell arteritis. A multicentre real-life study of 471 patients. Clin Exp Rheumatol 2023;41(4):829–36. 36377586 10.55563/clinexprheumatol/oqs8u9

[19] Tomelleri A Campochiaro C Farina N Mariotti L Baldissera E Grayson PC Effectiveness of a two-year tapered course of tocilizumab in patients with giant cell arteritis: A single-centre prospective study. Semin Arthritis Rheum 2023;59:152174. 36774660 10.1016/j.semarthrit.2023.152174 PMC9992325

[20] Muratore F Crescentini F Spaggiari L Pazzola G Casali M Boiardi L Aortic dilatation in patients with large vessel vasculitis: A longitudinal case control study using PET/CT. Semin Arthritis Rheum 2019;48(6):1074–82. 30424972 10.1016/j.semarthrit.2018.10.003

[21] García-Martínez A Arguis P Prieto-González S Espígol-Frigolé G Alba MA Butjosa M Prospective long term follow-up of a cohort of patients with giant cell arteritis screened for aortic structural damage (aneurysm or dilatation). Ann Rheum Dis 2014;73(10):1826–32. 23873881 10.1136/annrheumdis-2013-203322

[22] Christ L Seitz L Scholz G Sarbu AC Amsler J Bütikofer L Tocilizumab monotherapy after ultra-short glucocorticoid administration in giant cell arteritis: a single-arm, open-label, proof-of-concept study. Lancet Rheumatol 2021;3(9):e619–e26. 38287611 10.1016/S2665-9913(21)00152-1

[23] Muratore F Marvisi C Cassone G Ricordi C Boiardi L Mancuso P Treatment of giant cell arteritis with ultra-short glucocorticoids and tocilizumab: results from the extension of the TOPAZIO study. Rheumatology (Oxford, England) 2024. 10.1093/rheumatology/keae400 39150490

[24] Venhoff N Schmidt WA Bergner R Rech J Unger L Tony HP Safety and efficacy of secukinumab in patients with giant cell arteritis (TitAIN): a randomised, double-blind, placebo-controlled, phase 2 trial. Lancet Rheumatol 2023;5(6):e341–e50. 38251601 10.1016/S2665-9913(23)00101-7

[25] Hellmich B Sanchez-Alamo B Schirmer JH Berti A Blockmans D Cid MC EULAR recommendations for the management of ANCA-associated vasculitis: 2022 update. Ann Rheum Diseases 2024;83(1):30–47. 36927642 10.1136/ard-2022-223764

[26] Chalkia A Jayne D. ANCA-associated vasculitis-treatment standard. Nephrol Dial Transplant 2024;39(6):944–55. 37947275 10.1093/ndt/gfad237 PMC11210069

[27] Jayne DRW Merkel PA Schall TJ Bekker P. Avacopan for the Treatment of ANCA-Associated Vasculitis. NEJM 2021;384(7):599–609. 33596356 10.1056/NEJMoa2023386

[28] Palamidas DA Chatzis L Papadaki M Gissis I Kambas K Andreakos E Current Insights into Tissue Injury of Giant Cell Arteritis: From Acute Inflammatory Responses towards Inappropriate Tissue Remodeling. Cells 2024;13(5). 10.3390/cells13050430 PMC10930978 38474394

[29] Pugh D Karabayas M Basu N Cid MC Goel R Goodyear CS Large-vessel vasculitis. Nat Rev Dis Primers 2022;7(1):93. 34992251 10.1038/s41572-021-00327-5 PMC9115766

[30] Veroutis D Argyropoulou OD Goules AV Kambas K Palamidas DA Evangelou K Senescent cells in giant cell arteritis display an inflammatory phenotype participating in tissue injury via IL-6-dependent pathways. Ann Rheum Dis 2024;83(3):342–50. 38050005 10.1136/ard-2023-224467

[31] Fang C Du L Gao S Chen Y Chen Z Wu Z Association between premature vascular smooth muscle cells senescence and vascular inflammation in Takayasu's arteritis. Ann Rheum Dis 2024;83(11):1522–35. 38816066 10.1136/ard-2024-225630 PMC11503059

[32] Slart R. FDG-PET/CT(A) imaging in large vessel vasculitis and polymyalgia rheumatica: joint procedural recommendation of the EANM, SNMMI, and the PET Interest Group (PIG), and endorsed by the ASNC. Eur J Nucl Med Mol Imaging 2018;45(7):1250–69. 29637252 10.1007/s00259-018-3973-8 PMC5954002

[33] Pijl JP Nienhuis PH Kwee TC Glaudemans A Slart R Gormsen LC. Limitations and Pitfalls of FDG-PET/CT in Infection and Inflammation. Semin Nucl Med 2021;51(6):633–45. 34246448 10.1053/j.semnuclmed.2021.06.008

[34] Figueroa AL Abdelbaky A Truong QA Corsini E MacNabb MH Lavender ZR Measurement of arterial activity on routine FDG PET/CT images improves prediction of risk of future CV events. JACC Cardiovascular Imaging 2013;6(12):1250–9. 24269261 10.1016/j.jcmg.2013.08.006

[35] van der Geest KSM Treglia G Glaudemans A Brouwer E Sandovici M Jamar F Diagnostic value of [18F]FDG-PET/CT for treatment monitoring in large vessel vasculitis: a systematic review and meta-analysis. Eur J Nucl Med Mol Imaging 2021;48(12):3886–902. 33942141 10.1007/s00259-021-05362-8 PMC8484162

[36] Mehta NN Yu Y Saboury B Foroughi N Krishnamoorthy P Raper A Systemic and vascular inflammation in patients with moderate to severe psoriasis as measured by [18F]-fluorodeoxyglucose positron emission tomography-computed tomography (FDG-PET/CT): a pilot study. Arch Dermatol 2011;147(9):1031–9. 21576552 10.1001/archdermatol.2011.119 PMC3158301

[37] Kung BT Seraj SM Zadeh MZ Rojulpote C Kothekar E Ayubcha C An update on the role of (18)F-FDG-PET/CT in major infectious and inflammatory diseases. Am J Nucl Med Mol Imaging 2019;9(6):255–73. 31976156 PMC6971480

[38] Ćorović A Wall C Nus M Gopalan D Huang Y Imaz M Somatostatin Receptor PET/MR Imaging of Inflammation in Patients With Large Vessel Vasculitis and Atherosclerosis. J Am Coll Cardiol 2023;81(4):336–54. 36697134 10.1016/j.jacc.2022.10.034 PMC9883634

[39] Paravastu SS Theng EH Morris MA Grayson P Collins MT Maass-Moreno R Artificial Intelligence in Vascular-PET:: Translational and Clinical Applications. PET Clin 2022;17(1):95–113. 34809874 10.1016/j.cpet.2021.09.003

[40] Joy GM Arbiv OA Wong CK Lok SD Adderley NA Dobosz KM Prevalence, imaging patterns and risk factors of interstitial lung disease in connective tissue disease: a systematic review and meta-analysis. Eur Respir Rev 2023;32(167). 10.1183/16000617.0210-2022 PMC10032591 36889782

[41] Johnson SR Bernstein EJ Bolster MB Chung JH Danoff SK George MD 2023 American College of Rheumatology (ACR)/American College of Chest Physicians (CHEST) Guideline for the Screening and Monitoring of Interstitial Lung Disease in People with Systemic Autoimmune Rheumatic Diseases. Arthritis Care Res 2024;76(8):1070–82. 10.1002/acr.25347 PMC12646460 38973729

[42] Volkmann ER Kreuter M Hoffmann-Vold AM Wijsenbeek M Smith V Khanna D Dyspnoea and cough in patients with systemic sclerosis-associated interstitial lung disease in the SENSCIS trial. Rheumatology (Oxford, England) 2022;61(11):4397–408. 35150246 10.1093/rheumatology/keac091 PMC9629379

[43] Narváez J Aburto M Seoane-Mato D Bonilla G Acosta O Candelas G Screening criteria for interstitial lung disease associated to rheumatoid arthritis: Expert proposal based on Delphi methodology. Reumatologia clinica 2023;19(2):74–81. 35753951 10.1016/j.reumae.2021.12.003

[44] Khanna D Lin CJF Furst DE Goldin J Kim G Kuwana M Tocilizumab in systemic sclerosis: a randomised, double-blind, placebo-controlled, phase 3 trial. Lancet Respir Med 2020;8(10):963–74. 32866440 10.1016/S2213-2600(20)30318-0

[45] Chatzis LG Stergiou IE Goules AV Pezoulas V Tsourouflis G Fotiadis D Clinical picture, outcome and predictive factors of lymphoma in primary Sjögren's syndrome: results from a harmonized dataset (1981–2021). Rheumatology (Oxford, England) 2022;61(9):3576–85. 34940812 10.1093/rheumatology/keab939

[46] De Vita S Isola M Baldini C Goules AV Chatzis LG Quartuccio L Predicting lymphoma in Sjögren's syndrome and the pathogenetic role of parotid microenvironment through precise parotid swelling recording. Rheumatology (Oxford, England) 2023;62(4):1586–93. 36063040 10.1093/rheumatology/keac470 PMC10072883

[47] Pezoulas VC Goules A Kalatzis F Chatzis L Kourou KD Venetsanopoulou A Addressing the clinical unmet needs in primary Sjögren's Syndrome through the sharing, harmonization and federated analysis of 21 European cohorts. Comput Struct Biotechnol J 2022;20:471–84. 35070169 10.1016/j.csbj.2022.01.002 PMC8760551

[48] Goules AV Tzioufas AG. Lymphomagenesis in Sjögren's syndrome: Predictive biomarkers towards precision medicine. Autoimmun Rev 2019;18(2):137–43. 30572133 10.1016/j.autrev.2018.08.007

[49] Goules AV Chatzis L Pezoulas VC Patsouras M Mavragani C Quartuccio L Identification and evolution of predictors of Sjögren's disease-associated mucosa-associated lymphoid tissue lymphoma development over time: a case-control study. The Lancet Rheumatology. 2024;6(10):e693–e702. 39182505 10.1016/S2665-9913(24)00183-8

[50] Longhino S Chatzis LG Dal Pozzolo R Peretti S Fulvio G La Rocca G Sjögren's syndrome: one year in review 2023. Clinical and experimental rheumatology. 2023;41(12):2343–56. 38149515 10.55563/clinexprheumatol/255qsx

[51] Ruscitti P Allanore Y Baldini C Barilaro G Bartoloni Bocci E Bearzi P Tailoring the treatment of inflammatory rheumatic diseases by a better stratification and characterization of the clinical patient heterogeneity. Findings from a systematic literature review and experts' consensus. Autoimmunity reviews. 2024;23(7–8):103581. 39069240 10.1016/j.autrev.2024.103581

[52] Bowman SJ Fox R Dörner T Mariette X Papas A Grader-Beck T Safety and efficacy of subcutaneous ianalumab (VAY736) in patients with primary Sjögren's syndrome: a randomised, double-blind, placebo-controlled, phase 2b dose-finding trial. Lancet (London, England). 2022;399(10320):161–71. 34861168 10.1016/S0140-6736(21)02251-0

[53] Fisher BA Mariette X Papas A Grader-Beck T Bootsma H Ng WF Safety and efficacy of subcutaneous iscalimab (CFZ533) in two distinct populations of patients with Sjögren's disease (TWINSS): week 24 results of a randomised, double-blind, placebo-controlled, phase 2b dose-ranging study. Lancet (London, England) 2024;404(10452):540–53. 39096929 10.1016/S0140-6736(24)01211-X

[54] Dörner T Kaul M Szántó A Tseng JC Papas AS Pylvaenaeinen I Efficacy and safety of remibrutinib, a selective potent oral BTK inhibitor, in Sjögren's syndrome: results from a randomised, double-blind, placebo-controlled phase 2 trial. Ann Rheum Dis 2024;83(3):360–71. 37932009 10.1136/ard-2023-224691 PMC10894844

[55] St Clair EW Baer AN Ng WF Noaiseh G Baldini C Tarrant TK CD40 ligand antagonist dazodalibep in Sjögren's disease: a randomized, double-blinded, placebo-controlled, phase 2 trial. Nat Med 2024;30(6):1583–92. 38839899 10.1038/s41591-024-03009-3 PMC11186761

[56] Fusco FM Pisapia R Nardiello S Cicala SD Gaeta GB Brancaccio G. Fever of unknown origin (FUO): which are the factors influencing the final diagnosis? A 2005–2015 systematic review. BMC Infect Dis 2019;19(1):653. 31331269 10.1186/s12879-019-4285-8 PMC6647059

[57] Georgin-Lavialle S Fayand A Rodrigues F Bachmeyer C Savey L Grateau G. Autoinflammatory diseases: State of the art. Presse medicale (Paris, France: 1983). 2019;48(1 Pt 2):e25–e48. 30686513 10.1016/j.lpm.2018.12.003

[58] Gattorno M Hofer M Federici S Vanoni F Bovis F Aksentijevich I Classification criteria for autoinflammatory recurrent fevers. Ann Rheum Dis 2019;78(8):1025–32. 31018962 10.1136/annrheumdis-2019-215048

[59] Beck DB Ferrada MA Sikora KA Ombrello AK Collins JC Pei W Somatic Mutations in UBA1 and Severe Adult-Onset Autoinflammatory Disease. NEJM 2020;383(27):2628–38. 33108101 10.1056/NEJMoa2026834 PMC7847551

[60] Apostolidou E Skendros P Kambas K Mitroulis I Konstantinidis T Chrysanthopoulou A Neutrophil extracellular traps regulate IL-1β-mediated inflammation in familial Mediterranean fever. Ann Rheum Dis 2016;75(1):269–77. 25261578 10.1136/annrheumdis-2014-205958

[61] Skendros P Chrysanthopoulou A Rousset F Kambas K Arampatzioglou A Mitsios A Regulated in development and DNA damage responses 1 (REDD1) links stress with IL-1β-mediated familial Mediterranean fever attack through autophagy-driven neutrophil extracellular traps. J Allergy Clin Immunol 2017;140(5):1378–87.e13. 28342915 10.1016/j.jaci.2017.02.021

[62] Natsi AM Gavriilidis E Antoniadou C Papadimitriou E Papadopoulos V Tsironidou V IL-1β/DNA complex elevation distinguishes autoinflammatory disorders from autoimmune and infectious diseases. J Int Med 2024;296(3):298–301. 10.1111/joim.13809 38805484

[63] Vassilopoulos A Thomas K Vassilopoulos D. Infections in psoriatic arthritis: association with treatment. Ther Adv Musculoskelet Dis 2024;16:1759720x241289201. 10.1177/1759720X241289201 PMC11487508 39429971

[64] Zink A Manger B Kaufmann J Eisterhues C Krause A Listing J Evaluation of the RABBIT Risk Score for serious infections. Ann Rheum Dis 2014;73(9):1673–6. 23740236 10.1136/annrheumdis-2013-203341 PMC4145466

[65] Fragoulis GE Nikiphorou E Dey M Zhao SS Courvoisier DS Arnaud L 2022 EULAR recommendations for screening and prophylaxis of chronic and opportunistic infections in adults with autoimmune inflammatory rheumatic diseases. Ann Rheum Dis 2023;82(6):742–53. 36328476 10.1136/ard-2022-223335

[66] Kounatidis DC Papadimitropoulos V Avramidis K Plenga E Tsiara I Avgoustou E Pneumocystosis in a patient with rheumatoid arthritis on adalimumab therapy: a case-based review. Rheumatol Int 2024;44(2):363–7. 37851077 10.1007/s00296-023-05483-3

[67] Nurmohamed MT Kitas G. Cardiovascular risk in rheumatoid arthritis and diabetes: how does it compare and when does it start? Ann Rheum Dis 2011;70(6):881–3. 21450751 10.1136/ard.2010.145839

[68] Summers GD Metsios GS Stavropoulos-Kalinoglou A Kitas GD. Rheumatoid cachexia and cardiovascular disease. Nat Rev Rheumatol 2010;6(8):445–51. 20647995 10.1038/nrrheum.2010.105

[69] Toms TE Symmons DP Kitas GD. Dyslipidaemia in rheumatoid arthritis: the role of inflammation, drugs, lifestyle and genetic factors. Curr Vasc Pharmacol 2010;8(3):301–26. 19758115 10.2174/157016110791112269

[70] Kotenko SV Gallagher G Baurin VV Lewis-Antes A Shen M Shah NK IFN-lambdas mediate antiviral protection through a distinct class II cytokine receptor complex. Nat Immunol 2003;4(1):69–77. 12483210 10.1038/ni875

[71] Sheppard P Kindsvogel W Xu W Henderson K Schlutsmeyer S Whitmore TE IL-28, IL-29 and their class II cytokine receptor IL-28R. Nat Immunol 2003;4(1):63–8. 12469119 10.1038/ni873

[72] Hemann EA Gale M Jr Savan R. Interferon Lambda Genetics and Biology in Regulation of Viral Control. Front Immunol 2017;8:1707. 29270173 10.3389/fimmu.2017.01707 PMC5723907

[73] Galani IE Triantafyllia V Eleminiadou EE Koltsida O Stavropoulos A Manioudaki M Interferon-λ Mediates Non-redundant Front-Line Antiviral Protection against Influenza Virus Infection without Compromising Host Fitness. Immunity 2017;46(5):875–90.e6. 28514692 10.1016/j.immuni.2017.04.025

[74] Galani IE Rovina N Lampropoulou V Triantafyllia V Manioudaki M Pavlos E Untuned antiviral immunity in COVID-19 revealed by temporal type I/III interferon patterns and flu comparison. Nat Immunol 2021;22(1):32–40. 33277638 10.1038/s41590-020-00840-x

[75] Andreakos E Tsiodras S. COVID-19: lambda interferon against viral load and hyperinflammation. EMBO Mol Med 2020;12(6):e12465. 32333818 10.15252/emmm.202012465 PMC7267110

[76] Reis G Moreira Silva EAS Medeiros Silva DC Thabane L Campos VHS Ferreira TS Early Treatment with Pegylated Interferon Lambda for Covid-19. NEJM 2023;388(6):518–28. 36780676 10.1056/NEJMoa2209760 PMC9933926

[77] Andreakos E Zanoni I Galani IE. Lambda interferons come to light: dual function cytokines mediating antiviral immunity and damage control. Curr Opin Immunol 2019;56:67–75. 30399529 10.1016/j.coi.2018.10.007 PMC6541392

[78] Chrysanthopoulou A Kambas K Stakos D Mitroulis I Mitsios A Vidali V Interferon lambda1/IL-29 and inorganic polyphosphate are novel regulators of neutrophil-driven thromboinflammation. J Pathology 2017;243(1):111–22. 10.1002/path.4935 28678391

[79] Schrimpe-Rutledge AC Codreanu SG Sherrod SD McLean JA. Untargeted Metabolomics Strategies-Challenges and Emerging Directions. J Am Soc Mass Spectrom 2016;27(12):1897–905. 27624161 10.1007/s13361-016-1469-y PMC5110944

[80] Xu D Tang L Wang Y Pan J Su C. LC-MS-based rheumatoid arthritis serum metabolomics reveals the role of deoxyinosine in attenuating collagen-induced arthritis in mice. Heliyon 2024;10(10):e30903. 38778995 10.1016/j.heliyon.2024.e30903 PMC11108858

[81] Brinkmann V. Neutrophil Extracellular Traps in the Second Decade. J Innate Immun 2018;10(5–6):414–21. 29909412 10.1159/000489829 PMC6784051

[82] Papayannopoulos V. Neutrophil extracellular traps in immunity and disease. Nat Rev Immunol 2018;18(2):134–47. 28990587 10.1038/nri.2017.105

[83] Lande R Ganguly D Facchinetti V Frasca L Conrad C Gregorio J Neutrophils activate plasmacytoid dendritic cells by releasing self-DNA-peptide complexes in systemic lupus erythematosus. Sci Translat Med 2011;3(73):73ra19. 10.1126/scitranslmed.3001180 PMC3399524 21389263

[84] Hakkim A Fürnrohr BG Amann K Laube B Abed UA Brinkmann V Impairment of neutrophil extracellular trap degradation is associated with lupus nephritis. Proc Natl Acad Sci U S A 2010;107(21):9813–8. 20439745 10.1073/pnas.0909927107 PMC2906830

[85] Leffler J Martin M Gullstrand B Tydén H Lood C Truedsson L Neutrophil extracellular traps that are not degraded in systemic lupus erythematosus activate complement exacerbating the disease. J Immunol (Baltimore, Md: 1950). 2012;188(7):3522–31. 10.4049/jimmunol.1102404 22345666

[86] Khandpur R Carmona-Rivera C Vivekanandan-Giri A Gizinski A Yalavarthi S Knight JS NETs are a source of citrullinated autoantigens and stimulate inflammatory responses in rheumatoid arthritis. Sci Translat Med 2013;5(178):178ra40. 10.1126/scitranslmed.3005580 PMC3727661 23536012

[87] Palamidas DA Argyropoulou OD Georgantzoglou N Karatza E Xingi E Kapsogeorgou EK Neutrophil extracellular traps in giant cell arteritis biopsies: presentation, localization and co-expression with inflammatory cytokines. Rheumatology (Oxford, England) 2022;61(4):1639–44. 34260696 10.1093/rheumatology/keab505

[88] NaveenKumar SK Tambralli A Fonseca BM Yalavarthi S Liang W Hoy CK Low ectonucleotidase activity and increased neutrophil-platelet aggregates in patients with antiphospholipid syndrome. Blood 2024;143(12):1193–7. 38237140 10.1182/blood.2023022097 PMC10972706

[89] Brestoff JR. Full spectrum flow cytometry in the clinical laboratory. Int J Lab Hematol 2023;45(Suppl 2):44–9. 37211417 10.1111/ijlh.14098 PMC10330381

[90] Siouti E Salagianni M Manioudaki M Pavlos E Klinakis A Galani IE Notch signaling in adipose tissue macrophages prevents diet-induced inflammation and metabolic dysregulation. Eur J Immunol 2024;54(5):e2350669. 38339772 10.1002/eji.202350669

